# Shal/K_v_4 Channels Are Required for Maintaining Excitability during Repetitive Firing and Normal Locomotion in *Drosophila*

**DOI:** 10.1371/journal.pone.0016043

**Published:** 2011-01-17

**Authors:** Yong Ping, Girma Waro, Ashley Licursi, Sarah Smith, Dai-An Vo-Ba, Susan Tsunoda

**Affiliations:** Department of Biomedical Sciences, Colorado State University, Fort Collins, Colorado, United States of America; Columbia University, United States of America

## Abstract

**Background:**

Rhythmic behaviors, such as walking and breathing, involve the coordinated activity of central pattern generators in the CNS, sensory feedback from the PNS, to motoneuron output to muscles. Unraveling the intrinsic electrical properties of these cellular components is essential to understanding this coordinated activity. Here, we examine the significance of the transient A-type K^+^ current (I_A_), encoded by the highly conserved *Shal/K_v_4* gene, in neuronal firing patterns and repetitive behaviors. While I_A_ is present in nearly all neurons across species, elimination of I_A_ has been complicated in mammals because of multiple genes underlying I_A_, and/or electrical remodeling that occurs in response to affecting one gene.

**Methodology/Principal Findings:**

In *Drosophila*, the single *Shal/K_v_4* gene encodes the predominant I_A_ current in many neuronal cell bodies. Using a transgenically expressed dominant-negative subunit (DNK_v_4), we show that I_A_ is completely eliminated from cell bodies, with no effect on other currents. Most notably, DNK_v_4 neurons display multiple defects during prolonged stimuli. DNK_v_4 neurons display shortened latency to firing, a lower threshold for repetitive firing, and a progressive decrement in AP amplitude to an adapted state. We record from identified motoneurons and show that Shal/K_v_4 channels are similarly required for maintaining excitability during repetitive firing. We then examine larval crawling, and adult climbing and grooming, all behaviors that rely on repetitive firing. We show that all are defective in the absence of Shal/K_v_4 function. Further, knock-out of Shal/K_v_4 function specifically in motoneurons significantly affects the locomotion behaviors tested.

**Conclusions/Significance:**

Based on our results, Shal/K_v_4 channels regulate the initiation of firing, enable neurons to continuously fire throughout a prolonged stimulus, and also influence firing frequency. This study shows that Shal/K_v_4 channels play a key role in repetitively firing neurons during prolonged input/output, and suggests that their function and regulation are important for rhythmic behaviors.

## Introduction

Rhythmic behaviors, such as walking and breathing, are the consequence of the proper interplay of central pattern generators (CPGs) in the CNS, sensory input from the PNS, and motoneurons projecting to muscles. How these components are coordinated to produce proper motor output, however, is still a mystery. *Drosophila* has served as a model system for studying rhythmic behaviors, such as locomotion, and much is known about motoneurons and their targets [1]. Less is known about the intrinsic electrical properties of these different cellular components and how they contribute to rhythmic behaviors. Here, we examine the significance of the transient A-type K^+^ current (I_A_) encoded by *Shal/K_v_4* in neuronal firing patterns and repetitive behaviors.

Identifying the role of I_A_ has been complicated in mammals since I_A_ is often encoded by multiple genes. For example, I_A_ in pyramidal neurons of the mouse visual cortex has been shown to be encoded by *K_v_4.2*, *K_v_4.3*, and *K_v_1.4* [2]. With a paucity of specific channel blockers, studies have been unable to completely abolish I_A_. For example, even “Shal-specific” toxins, like PaTx2 and Phrixotoxin, are unable to completely block Shal/K_v_4 currents heterologously expressed in *Xenopus* oocytes or in neurons [3], [4]. Genetic mutations have also been unsuccessful at eliminating I_A_, usually due to multiple genes underlying I_A_, and/or homeostatic electrical remodeling [2], [5]–[10].

In *Drosophila*, only two genes, *Shaker/K_v_1* and *Shal/K_v_4*, encode A-type K^+^ currents [11]–[16]. While mammals contain multiple *K_v_1* and *K_v_4* genes, *Drosophila* contains only one of each. *Drosophila Shaker/K_v_1* has been shown to underlie the major A-type current in muscle cells [13], [15]–[20], while in neurons, these channels are restricted to axons and nerve terminals [21], [22]. In contrast, Shal/K_v_4 channels are localized exclusively to somato-dendritic compartments of neurons [23], [24], and have been shown to carry the predominant I_A_ current in the cell bodies of most *Drosophila* neurons [3], [18], [25]–[27]. In addition to different expression and subcellular localization patterns, Shaker/K_v_1 and Shal/K_v_4 channels also display distinct biophysical properties. Most notably, the rapid inactivation of Shaker/K_v_1 currents is clearly voltage-dependent, while Shal/K_v_4 current inactivation, even if variable from cell to cell, is usually voltage-independent [13], [15]–[20], [25], [28]. Also distinct is the significantly more hyperpolarized voltage-operating range of Shal/K_v_4 channels [3], [25], [26], [28], making them more similar to the classic A-current described by Connor and Stevens (1971) [29]. Shal/K_v_4 channels, unlike Shaker/K_v_1 channels, are also highly conserved across species with respect to sequence identity, subcellular localization, and biophysical properties. Little, however, is known about the role Shal/K_v_4 plays in neuronal firing and rhythmic behaviors.

Here, we generate transgenic *Drosophila* lines expressing a dominant-negative K_v_4 subunit (DNK_v_4). We show that DNK_v_4 completely eliminates I_A_, without affecting other K^+^ and Na^+^ currents. Thus, we are able to examine K_v_4/I_A_ function during neuronal firing, and in the absence of any cell intrinsic electrical remodeling. Most notable is the critical role of K_v_4/I_A_ during repetitive firing. DNK_v_4 motoneurons fire APs with progressively smaller amplitudes, reaching an adapted, non-excitable state. We show that larval crawling, and adult climbing and grooming, all behaviors that rely on repetitive firing, are defective in the absence of Shal/K_v_4 function. Our results strongly suggest that Shal/K_v_4 is required for normal motoneuron firing, which drives rhythmic behaviors.

## Results

### Transgenic Lines Expressing a Dominant-Negative K_v_4 Subunit (DNK_v_4) Display Complete Elimination of Somatic I_A_

To examine the functional significance of K_v_4 channels in *Drosophila* neurons, we generated transgenic lines expressing a dominant-negative K_v_4 channel subunit, DNK_v_4. Modeled after a similar mutation in mammalian K_v_4.2 that was shown to render channels non-functional [30], DNK_v_4 contains a phenylalanine substituted for a tryptophan residue in the pore loop of the channel. The *DNK_v_4* sequence was expressed under the control of the *upstream activating sequence* (*UAS; UAS-DNK_v_4*) so that we could employ the *UAS/Gal4* system to target DNK_v_4 to specific tissues, and even specific cell types. The DNK_v_4 also contains an N-terminal hemagglutinin (HA) epitope tag for expression and localization studies. Since *K_v_4* is normally expressed throughout the nervous system, we first used the neuron-specific *elav-Gal4* transgene to drive expression of *DNK_v_4* (*elav-Gal4:UAS-DNK_v_4*) in all neurons; indeed, DNK_v_4 showed co-localization with GFP-Shal channels in cell bodies and puncta along neuronal processes (data not shown).

**Figure 1:**
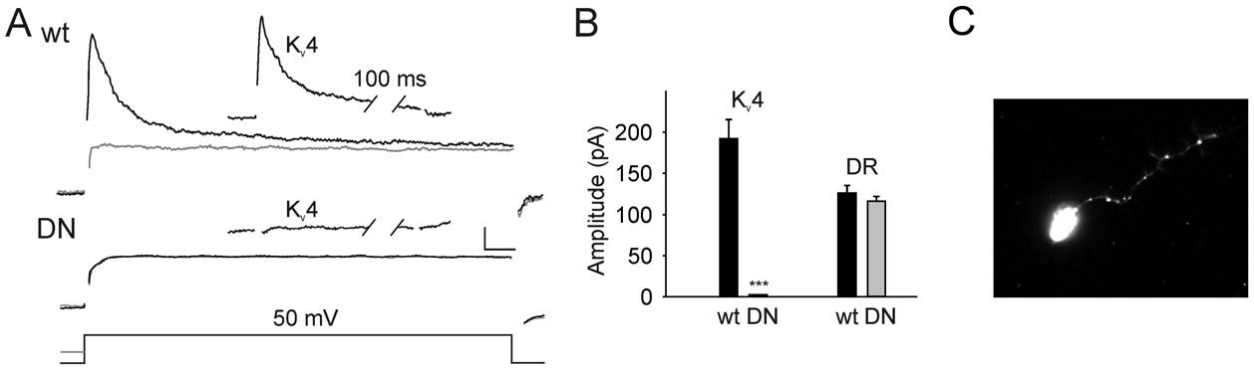
DNK_v_4 Neurons Display Complete Elimination of I_A_. *A*, Shown are representative voltage-clamp recordings from a wild-type (wt) and DNK_v_4 (DN) neuron in response to a voltage jump to +50 mV. Total whole-cell K^+^ current is elicited with a 500 ms prepulse of −125 mV (black trace). With a prepulse of −45 mV, K_v_4 is completely inactivated and the total delayed rectifier current remains (grey trace). Insets show the K_v_4 current trace obtained by subtracting the delayed rectifier current trace from the total K^+^ current trace. *B*, Average peak K_v_4 and delayed rectifier (DR) currents shown for wt and DN neurons; there is no significant difference in amplitude of the delayed rectifier currents (DR) remaining wild-type and DNK_v_4 neurons (N = 15 for each genotype). Scale bars represent 50 pA and 10 ms. *C*, Single embryonic neuron expressing the HA-tagged DNK_v_4 subunit immunostained for HA. DNK_v_4 appears to be localized to the cell body and in puncta along neuronal processes.

To test whether expression of *DNK_v_4* does indeed result in a dominant-negative effect on the I_A_ current, we performed voltage-clamp recordings from embryonic neurons in culture. In these neurons, I_A_ currents vary, especially in their inactivation rates, but are all completely eliminated with a deletion of the *K_v_4* gene [25]. We show, similarly, that I_A_ is completely abolished in DNK_v_4 neurons, with only the delayed rectifier current remaining (Figure 1A). To ensure that these results were indeed due to the expression of *DNK_v_4*, and not from insertion of the transgene in the chromosome, we examined two independently generated transgenic lines. Both lines showed a complete elimination of I_A_ (data not shown).

**Figure 2:**
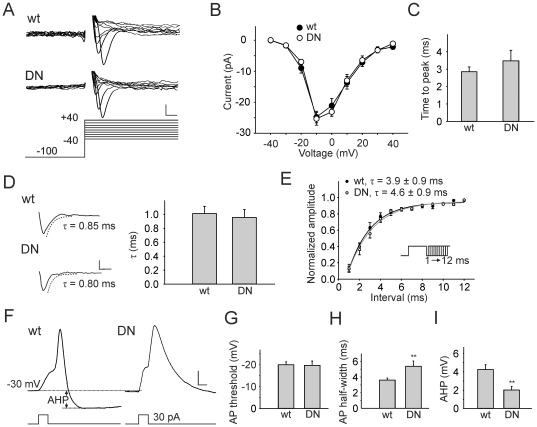
K_v_4 Modulates Repolarization of the AP and AHP, with No Effect on the Sodium Current. *A–D*, Representative Na^+^ current traces and analyses. Na^+^ currents were recorded in response to voltage jumps to potentials from −40 to +40 mV, in 5 mV increments, from a holding potential of −100 mV. For clarity, only part of the traces, corresponding to the first 18 ms of the voltage jump, in 10 mV increments are shown (*A*). There were no significant differences, between wild-type (wt) and DNK_v_4 (DN) neurons, in their I-V curves (*B*; N = 18 for wt, N = 9 for DN), times to peak current during a voltage jump to −10 mV (*C*; N = 19 for wt, N = 9 for DN, p>0.05), and time courses of inactivation during a voltage jump to −10 mV (*D*; N = 18 for wt, N = 9 for DN, p>0.05). Scale bars represent 10 pA and 2 ms. *E*, Recovery from inactivation for Na^+^ currents was examined in a series of paired voltage jumps, each up to −10 mV for 10 ms. The second voltage jump was initiated 1 to 12 ms, in 1 ms increments, following the first voltage jump (inset). Amplitude of the second Na^+^ current is expressed as a normalized fraction of the first, plotted time interval between jumps. Data were fit with a single exponential: f(t) = C+A*exp(-t/τ_rec_). An averaged plot is shown. There was no significant difference between τ_rec_ values from wild-type and DNK_v_4 neurons (τ_rec_ = 3.9±0.9, N = 12 for wt; τ_rec_ = 4.6±0.8, N = 8 for DN, p>0.05). *F*, Single AP elicited from representative wt and DN neurons using a 5 ms injection of 30 pA (traces shown are averages of 7 consecutive recordings for each). Since AP thresholds were relatively high, we depolarized the cell to about −30 mV with a prepulse (∼30 pA, 50 ms) before evoking APs. *G*, AP thresholds were defined as the voltage at which dV/dt reached 2 V/s; no significant difference in the AP threshold was observed between wild-type and DNK_v_4 neurons (N = 11 for wt, N = 13 for DN, p>0.05). *H*, AP half-widths were measured half-way between the baseline (just before the AP, see dashed line) and the AP peak. AP half-widths were significantly larger in DNK_v_4 neurons (N = 11 for wt, N = 13 for DN). *I*, AHPs were measured from the baseline voltage just before the AP (see horizontal dashed line, top) to the negative peak following the AP (see horizontal dashed line, bottom), as indicated. AHPs were significantly decreased in DN neurons (N = 11 for wt, N = 13 for DN). Scale bars represent 5 mV, 5 ms. For additional characterization of the I_Na_, see Figure S1.

We found no compensatory current expressed in DNK_v_4 neurons. No transient A-type current was seen in any neuron (N>50), and there were no differences between the delayed rectifier currents of wild-type and DNK_v_4 neurons (Figure 1). We also examined the voltage-dependent Na^+^ current present, and found no difference in I_Na_ amplitude, voltage-dependent I-V relation, times to peak current, rates of inactivation, rate of recovery from inactivation, or steady-state inactivation properties (Figure 2A–E, Figure S1). Thus, the loss of K_v_4 function did not result in any detectable electrical remodeling in these neurons. We were, therefore, able to examine how the complete and selective loss of I_A_/K_v_4 affects neuronal firing patterns.

### K_v_4 Channels Regulate Latency, Threshold, and Interspike Interval of Repetitive Firing Sequences in Neurons

Resting potentials of wild-type and DNK_v_4 neurons were similar at 61+/−0.2 mV (N = 22) and 61+/−0.4 mV (N = 24), respectively, suggesting that K_v_4 channels do not contribute to resting conductances. We first investigated the role K_v_4 plays in single action potentials (APs) by applying 5 ms current injections of 30 pA to neurons; since AP thresholds were relatively high, we depolarized the cell to about −30 mV before evoking APs. Single APs from DNK_v_4 neurons showed no difference in AP threshold compared with wild-type neurons (Figure 2F,G). Significant differences, however, were observed in AP durations and afterhyperpolarizations (AHPs). DNK_v_4 neurons had much broader APs with little to no AHPs, compared to wild-type (Figure 2F,H–I). These results show that K_v_4 channels play a significant role in the repolarization of APs, and repolarization of the membrane following APs.

**Figure 3:**
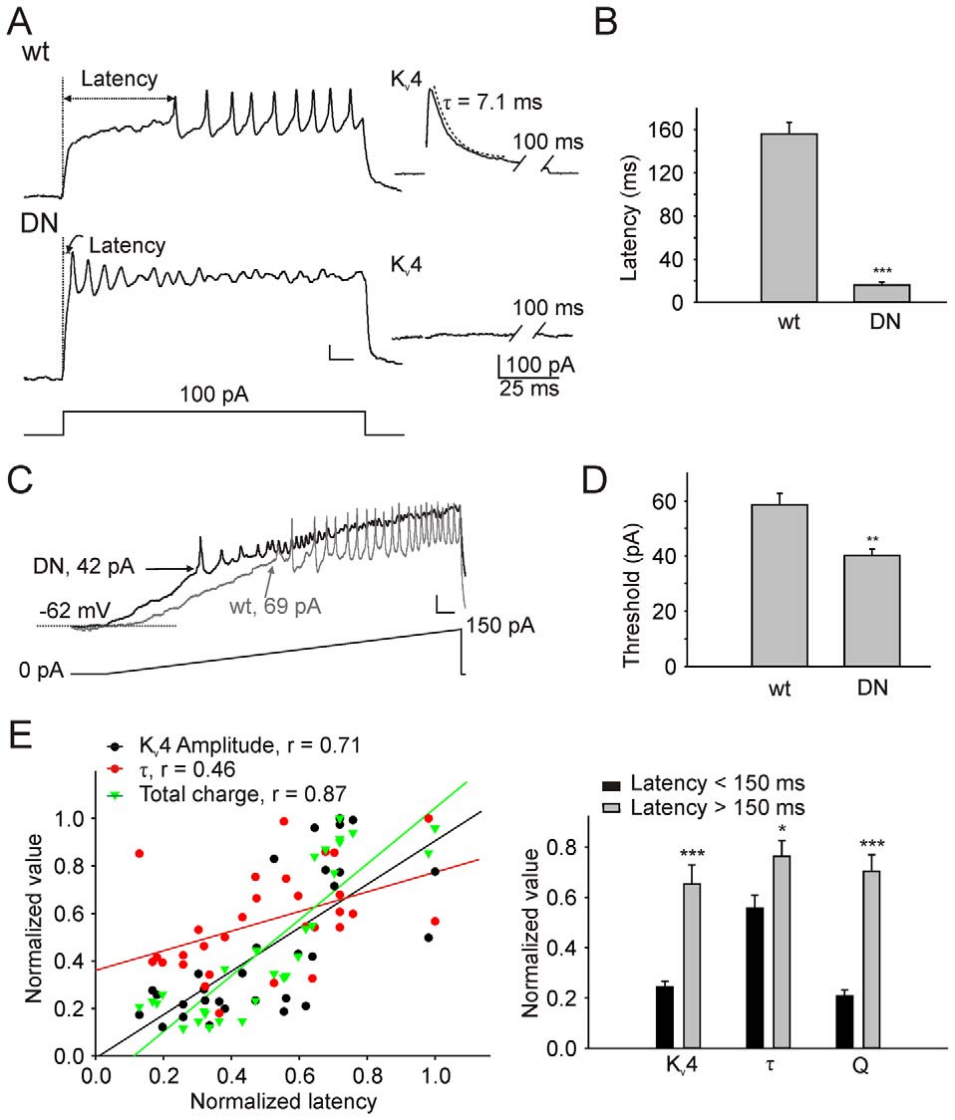
K_v_4 Regulates the Latency to the First AP. *A*, Representative current-clamp recording from a wild-type (wt) and DNK_v_4 (DN) neuron in response to 500 ms current injection of 100 pA. Insets (right) show the K_v_4 current recorded in voltage-clamp mode (generated using the protocol described in Fig. 1) from the same cell. *B*, Latency to the first AP peak is dramatically decreased in DN neurons (156.0±20.9 ms, N = 30 for wt; 16.2±2.6 ms, N = 20 for DN). *C*, Thresholds for repetitive AP firing in wild-type (black trace) and DNK_v_4 (grey trace) neurons as measured during a representative recording in response to a ramp injection protocol from 0 to 150 pA, over 1 sec. The dotted line shows the resting membrane potential, arrows indicate thresholds for AP firing. *D*, The average threshold for repetitive AP firing is significantly reduced in DN neurons (40.1±2.3 pA, N = 12), compared with wild-type (58.5±4.0 pA, N = 8). *E*, For each wild-type neuron (N = 30), latency to the first AP (in response to a 100 pA current injection) was measured, then, in voltage-clamp mode the K_v_4 current was isolated and analyzed. Left, K_v_4 amplitude, fast inactivation time constant (τ), and total charge (Q) were plotted against the latency to first AP peak; all values were normalized to the largest value in the group. Fast τ was determined from the best-fit single exponential function to the first 30 ms after the peak of the of the K_v_4 current trace (see A inset). K_v_4 amplitude, τ, and especially Q, were found to positively correlate with latency, exhibiting correlation coefficients (r) of 0.71, 0.46, and 0.87, respectively. Right, K_v_4 amplitude, τ, and Q are all increased with longer latencies (>150 ms versus <150 ms). Scale bars represent 10 mV and 40 ms. Additional experiments were performed to examine how pre-pulse potentials regulating the availability of K_v_4 channels also regulate latency to AP firing (Figure S2).

We then used longer pulses (500 ms) of current injection and compared firing patterns in wild-type and DNK_v_4 neurons. While wild-type neurons displayed clear latencies to firing, one of the most pronounced differences in DNK_v_4 neurons was the near absence of a delay to the first AP in all DNK_v_4 neurons (Figure 3A–B, 4A). Consistent with this decreased latency to AP firing, we also found that DNK_v_4 neurons had a lower threshold for inducing repetitive firing. This was evident in the amount of current injection required to induce repetitive firing during both prolonged stimuli; wild-type neurons consistently required larger current injections to induce firing (Figure 3C–D, 4A). When the current injection was ramped from 0 to 150 pA, firing also induced earlier in DNK_v_4 neurons, compared with wild-type. The requirement for higher stimuli to induce repetitive firing was not due to a difference in resting potential since membrane potentials at rest were not significant different between wild-type and DNK_v_4.

Since K_v_4 currents in wild-type embryonic neurons vary, especially in their inactivation rates [25], [31], we investigated whether these K_v_4 current properties correlated to differences in latency to the first AP. For each neuron, a current-clamp recording was performed to determine the delay to the first AP in response to a 100 pA current injection. Then, recording was switched to voltage-clamp mode and tetrodotoxin was added to the bath to reveal only the voltage-dependent K^+^ currents present. The K_v_4 current was isolated by using a pre-pulse protocol (see Figure 1A). K_v_4 amplitude, fast inactivation rate (τ_fast_), and total charge carried were normalized and plotted against the latency to the first AP recorded in current-clamp mode. Indeed, delay to the first AP showed a positive correlation with each of these K_v_4 current properties (Figure 3E, left). When we divided cells into groups of shorter (<150 ms) and longer (>150 ms) AP latencies, corresponding K_v_4 current amplitude, τ_fast_, and charge were significantly larger in cells with longer latencies (Figure 3E, right). We also used various pre-pulse protocols to regulate the membrane potential, and therefore the availability of K_v_4 channels for activation, and show that this correlates with the latency to the first AP (see Figure S2). These results all show that K_v_4 channels regulate the latency to repetitive firing, and that regulation of K_v_4 current properties can dictate this delay.

**Figure 4:**
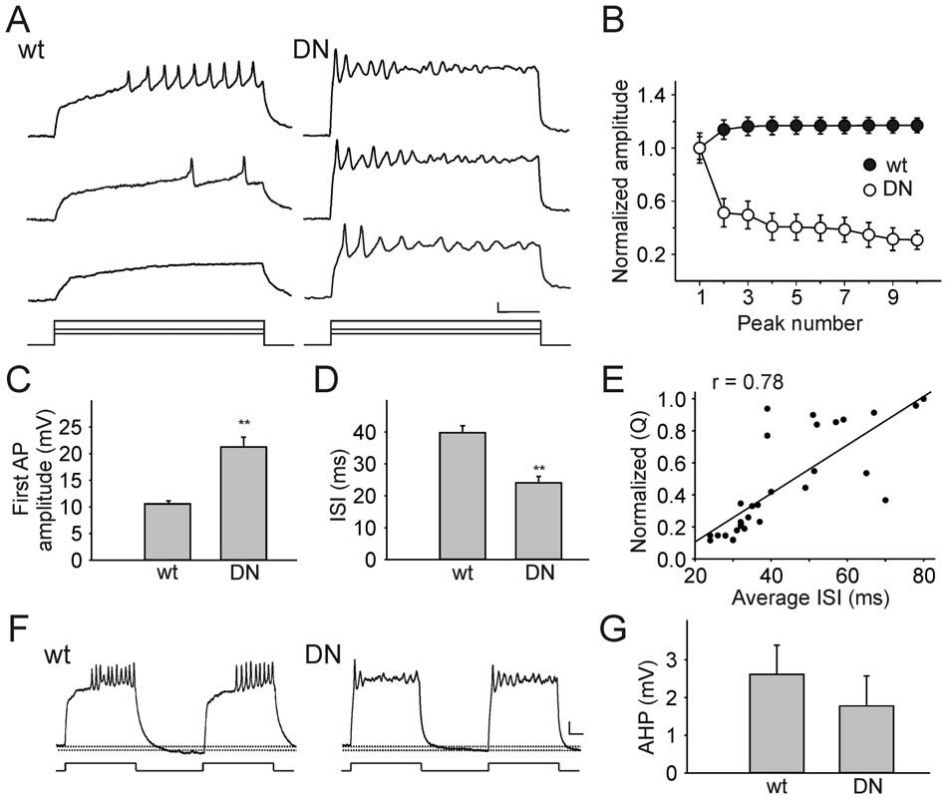
K_v_4 is Required for Maintaining Excitability in *Drosophila* Neurons. *A*, Representative firing patterns recorded with current injections of 40, 60, and 100 pA from wild-type (wt) and DNK_v_4 (DN) neurons. With increasing current injections, wt neurons display a reduction in the delay to the first AP and an augmentation in AP firing frequency, maintaining excitability for the duration of the 500 ms stimulus. DN neurons display little delay to the first AP, and subsequent peaks decrease in amplitude, compared with wt. *B*, Plotted are amplitudes of the first 10 peaks, representing APs or graded potentials, normalized to the first AP (N = 30 for wt, N = 20 for DN); a pronounced adaptation of peaks in DN neurons is observed. *C*, The average amplitude of the first AP in DN neurons (N = 20) is significantly greater than wt (N = 30). *D*, The interspike interval (ISI), measured as the time between the peaks of the first and second APs, is significantly decreased in DN neurons (N = 20) compared to wt (N = 30). *E*, Current-clamp recording was performed (100 pA, 500 ms), followed by a voltage-clamp recording to isolate the K_v_4 current, as described in Figure 1A. Shown is the normalized charge carried by the K_v_4 current plotted against the average interspike interval between each AP fired during the stimulus. A positive correlation (r = 0.78, N = 15) is seen between K_v_4 current charge and average ISI. *F–G*, Shown are voltage responses of representative wt and DN neurons to a pair of 500 ms current injections of 100 pA, with an interval of 500 ms. The AHP that follows each stimulus “burst” is measured as the hyperpolarization beyond the membrane potential before the stimulus, as indicated by the doted lines. No significant difference in this “interburst” AHP was seen between wild-type and DNK_v_4 neurons (N = 9 for wt, N = 11 for DN) (G). Scale bars represent 10 mV and 100 ms.

During prolonged current injections, we also observed that the first AP in DNK_v_4 neurons was consistently larger in amplitude (Figure 4A,C). These results suggest that K_v_4 channels function in the repolarization of the AP (as seen with single APs). In addition, the interspike interval (ISI) measured between the first and second APs of DNK_v_4 neurons was also significantly shortened (Figure 4A,D), suggesting that K_v_4 functions also at sub-threshold potentials, regulating firing frequency. We next examined if there is indeed a correlation between K_v_4 current and firing frequency in wild-type neurons. To do this, we applied a 500 ms current injection to wild-type neurons in current-clamp mode, measured the average ISI between APs during the stimulus, then switched to voltage-clamp mode to isolate the K_v_4 current present. For each cell, the total charge carried by the K_v_4 current was normalized and plotted against the average ISI recorded in that same cell. Indeed, there is a strong positive correlation between the K_v_4 current carried and the ISI (Figure 4E), indicating that K_v_4 and regulation of K_v_4 properties can set the firing frequency. Altogether, our results show that K_v_4 regulates the delay to first firing, the repolarization and AHP of each AP, and subsequent firing frequency during prolonged stimuli.

### K_v_4 is Required for Maintaining Excitability During Repetitive Firing

After a delay to the first AP firing, wild-type neurons maintain excitability throughout a 500-ms stimulus, firing repeatedly with no decrement in AP size until the end of the stimulus (Figure 4A). In contrast, DNK_v_4 neurons fire one or two initial APs, followed by subsequent depolarizing peaks with decreasing amplitude (Figure 4A,B). This adaptation, or accommodation, becomes faster with increasing amounts of current injection. The relatively more depolarized membrane potential of DNK_v_4 neurons during the current stimulus (see Figure 4A) suggests that without K_v_4 channel function, the membrane potential is not propertly repolarized. The broadened durations and reduced AHP of single APs in DNK_v_4 neurons (Figure 2F, I) suggest that K_v_4 channels repolarize the membrane after AP firing. It is likely that this repolarization of the membrane is required for Na^+^ channels to recover from inactivation. Thus, without proper recovery of Na^+^ channels after the first or second AP, the neuron loses the ability to fire again and again.

**Figure 5:**
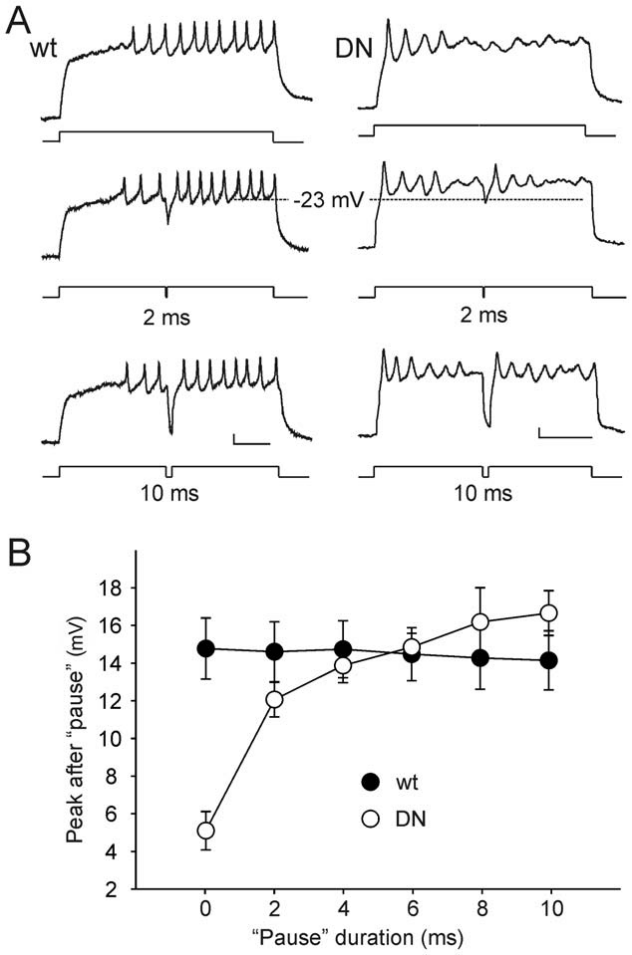
Membrane Repolarization in DNK_v_4 Neurons Restores Excitability. *A*, Shown are representative wild-type (wt) and DNK_v_4 (DN) voltage responses to a 500 ms stimulus of 100 pA either uninterrupted (top), or interrupted with a 2 ms (middle) or 10 ms (bottom) “pause” during which no current stimulus is applied; the longer the pause, the more repolarized the membrane during the pause. Note that while excitability is lost during the uninterrupted current stimulus of the DN neuron, the 2 ms pause repolarized the membrane to ∼23 mV and restored excitability: AP is seen following the pause. Excitability was unaffected wt. *B*, The same experiment, as described in (*A*), was performed with pauses of 0, 2, 4, 6, 8, and 10 ms for wt and DN neurons (N = 11 for wt, N = 13 for DN). In DN neurons, average amplitudes of the AP following the pause (Peak after Pause) increased with the duration of the pause given, while AP amplitudes in wild-type remained constant. Scale bars represent 10 mV and 100 ms.

To test whether membrane repolarization is indeed the critical factor to restoring excitability to DNK_v_4 neurons, we introduced a “pause” in the middle of current injections that allow for transient membrane repolarization. Indeed, AP firing in DNK_v_4 neurons was restored following the pause (Figure 5A). In addition, the extent to which excitability was restored correlated directly with the duration of the pause and magnitude of repolarization that was introduced (Figure 5B). Together, our results suggest that K_v_4 repolarizes the membrane during prolonged stimuli, and that this is critical for maintaining excitability during repetitive firing. Without sufficient membrane repolarization, there is less or no recovery of Na^+^ channels from inactivation, and no further AP firing. Since no changes were observed in I_Na_ properties, the effects of DNK_v_4 are likely to be due exclusively to the loss of K_v_4 function.

### DNK_v_4 Motoneurons Display Similar Defects in Repetitive Firing

**Figure 6:**
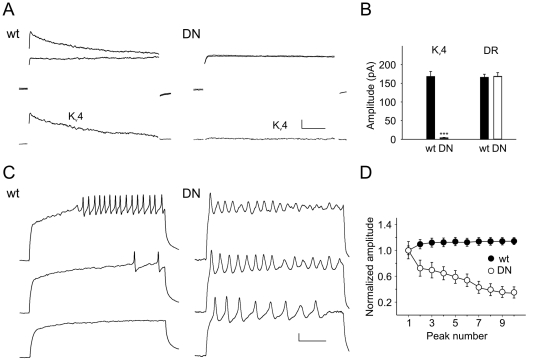
DNK_v_4 Motoneurons Display Similar Defects in Repetitive Firing. *A*, *Top*, Shown are representative voltage-clamp recordings from a wild-type neuron (wt) and a DNK_v_4 identified aCC/RP2 motoneuron (DN) in response to a voltage jump to +50 mV. Total whole-cell K+ current is elicited with a 500 ms prepulse of −125 mV (top trace). With a prepulse of −45 mV, K_v_4 is completely inactivated and the total delayed rectifier current remains (bottom trace). *Bottom*, The isolated K_v_4 current trace is obtained, and shown, by subtracting the delayed rectifier current trace from the total K^+^ current trace. Scale bars represent 50 pA and 25 ms. *B*, Average peak K_v_4 and delayed rectifier (DR) currents shown for wt and DN motoneurons; no significant difference in amplitudes is seen (N = 17 for wt, N = 11 for DN). *C*, Representative firing patterns recorded with current injections of 40, 60, and 100 pA from wt and DN motoneurons. Note that DN motoneurons display a shortened delay to the first AP, a greater current injection required for inducing repetitive firing, an increased firing frequency, and adaptation in repetitive firing seen at higher current injections, when compared with wt motoneurons. Scale bars represent 10 mV and 100 ms. *D*, Plotted are amplitudes of the first 10 peaks, representing APs or graded potentials, normalized to the first AP (N = 9 for each genotype); similar to Figure 4, a pronounced adaptation is observed in DN motoneurons. All motoneurons are identified by the expression of CD8-GFP driven by *RRa-Gal4*.

Since reliable, repetitive firing is especially important for motoneurons during rhythmic behaviors, we hypothesized that K_v_4 channels might play a significant role in maintaining continuous firing patterns in motoneurons. To test this hypothesis, we examined K_v_4 channel function in identified motoneurons. We used the *RRa-Gal4* transgene to drive expression of *UAS-mCD8-GFP* specifically in aCC and RP2 motoneurons [32], [33]; M. Fujioka, personal communication). Similar voltage-clamp and current-clamp studies, as described above, were performed with individual motoneurons. Resting potentials were similar in these motoneurons to those recorded from the general neuronal population, however, cells were larger, with capacitances of ∼5.5 pF, compared with ∼2.4 pF for neurons in the general population. We found that motoneurons displayed K_v_4 currents and firing patterns similar to other neurons, but with much less variability from cell to cell, as expected (Figure 6).

When expression of *UAS-DNK_v_4* was also driven in aCC and RP2 motoneurons, the entire I_A_ current was also removed with no effect on the delayed rectifier current present (Figure 6A–B). These results show that the entire somatic I_A_ in these motoneurons is indeed encoded by *Shal/K_v_4*. Loss of K_v_4 function in these motoneurons had similar effects as described for the general neuronal population. For example, repetitive firing in DNK_v_4 motoneurons was always induced with a lower current stimulus than wild-type motoneurons (Figure 6C). Latency to the first AP was regulated by K_v_4 channels, as seen with other neurons. In contrast to wild-type motoneurons, which displayed a very stereotypical delay from cell to cell, DNK_v_4 motoneurons displayed virtually no delay to firing (Figure 6C). Initial APs in DNK_v_4 motoneurons were larger than wild-type motoneurons, and firing frequency at any given stimulus was also increased in DNK_v_4 motoneurons (Figure 6C).

Finally, DNK_v_4 motoneurons also showed a loss of excitability during prolonged stimuli. Without K_v_4 channel function, firing frequency was increased, but AP amplitudes progressively decreased during the stimulus as shown in Figure 6D, reaching an adapted state especially with large current injections (Figure 6C–D). Although DNK_v_4 motoneurons appeared to display less adaptation than neurons in the general population, they clearly adapted more easily than wild-type motoneurons.

**Figure 7:**
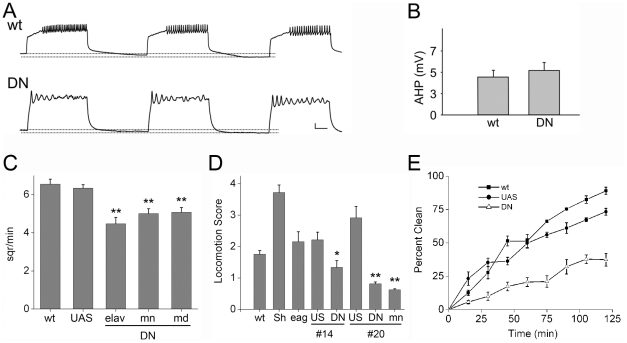
Loss of K_v_4 Function Affects Repetitive Firing in Motoneurons, Larval and Adult Locomotion, and Grooming. *A*, Shown are voltage responses of representative wild-type (wt) and DNK_v_4 (DN) motoneurons, identified by *RRa-Gal4:UAS-CD8-GFP*, to repeated 500 ms pulses of 100 pA current injections, with intervals of 500 ms; this protocol mimics stimuli during fictive larval locomotion (see text). Note the loss of latency to firing, and loss of excitability for repetitive firing until the end of each stimulus, in the DN motoneuron. The AHP that follows each stimulus “burst” is measured as the hyperpolarization beyond the membrane potential before the stimulus, as indicated by the dotted lines. Scale bars represent 10 mV and 100 ms. *B*, Average amplitudes of the interburst AHP are shown; no significant difference was observed between wt and DN motoneurons (N = 5 for wt, N = 6 for DN). *C*, Crawling speed of individual third instar larvae was measured as the number of 0.5×0.5 cm squares crossed on an agarose plate in a five minute period. Averages from *UAS-DNK_v_4* (UAS), *elav-Gal4:UAS-DNK_v_4* (elav), *c164-Gal4:DNK_v_4*, and *109(80)-Gal4:DNK_v_4* (md) larvae are shown; 14–15 larvae from each genotype were tested. Note that all DN genotypes, whether driven in the entire nervous system (elav), in all motoneurons (mn), or in all multi-dendritic sensory neurons (md), displayed significantly slower crawling speeds from wt and the UAS background stock. *D*, Adult locomotion was tested in a climbing assay on adult wt, *Sh^KS133^* (Sh), eag^1^ (eag), *UAS-DNK_v_4#14* (US, #14), *elav-Gal4:UAS-DNK_v_4#14* (DN, #14), *UAS-DNK_v_4#20* (US, #20), *elav-Gal4:UAS-DNK_v_4#20* (DN, #20), and *c164-Gal4: DNK_v_4#20* (mn, #20) flies (see Materials and Methods); each fly was given one point for every two tubes they climbed out of. The mean score of flies from each group was noted; this was then repeated for ten groups for each genotype with averages shown. All DN stocks, driven in the whole nervous system or in all motoneurons, displayed significantly lower scores than their respective background stocks. (E) Adult flies were tested for grooming (see Materials and Methods). Average percent clean flies at time points from 0 to 120 minutes are shown; three to four groups were examined for each time point. Time-courses for wt, *UAS-DNK_v_4#14* (UAS), and *elav-Gal4;UAS-DNK_v_4#14* (DN) flies are shown.

To examine how the loss of K_v_4 function in motoneurons would likely affect their motor output, we applied repeated 500 ms current injections, at frequency of 1 Hz, to simulate the synaptic input motoneurons would likely receive during crawling, as previously described [34]. During these repeated depolarizations, wild-type neurons displayed bursts of firing which began after a constant delay and then continued until the end of the burst (Figure 7A). In contrast, DNK_v_4 motoneurons fired bursts with no delay to the first AP, but adapted easily and did not fire continuously until the end of the burst like wild-type (Figure 7A). Note that the AHP between bursts was still present in DNK_v_4 motoneurons (Figure 7A), and indistinguishable from wild-type (Figure 7B), consistent with the report that this AHP is due to the Na^+^-K^+^ pump [34]. K_v_4, in contrast, is responsible for repolarization of the membrane after AP firing during repetitive firing, and it is this membrane repolarization that is required for maintaining excitability during bursts of AP firing.

### DNK_v_4 Flies Display Defects in Rhythmic Behaviors that Depend on Repetitive Firing

Since DNK_v_4 motoneurons displayed substantial defects in repetitive firing, we investigated whether these defects would be translated to behaviors that involve repetitive or rhythmic movement. In larvae, we performed a crawling assay, similar to a previous study [35]. We found that third instar *elav-Gal4:UAS-DNK_v_4* larvae crawled with a significantly slower speed, compared with both wild-type and the *UAS-DNK_v_4* background stock (Figure 7C). To determine if K_v_4 function in motoneurons contributes to this defect, we used the *c164-Gal4* transgene to drive expression of *UAS-DNK_v_4* only in motoneurons. Indeed, these larvae (*c164-Gal4:UAS-DNK_v_4*) exhibited crawling speeds significantly slower than wild-type and background controls, and equally impaired as larvae with DNK_v_4 was expressed throughout the nervous system (Figure 7C).

We then extended these studies to adult flies. We used a previously developed assay to evaluate the ability of adult flies to climb against gravity [35], [36]. We compared wild-type, two different transgenic *elav-Gal4:UAS-DNK_v_4* lines, the corresponding *UAS-DNK_v_4* background stocks, and two other K^+^ channel mutants, *Sh^KS133^* and *eag^1^* (Figure 7C). We found that both *elav-Gal4:UAS-DNKv4* transgenic lines displayed significantly lower climbing scores than wild-type and background stocks (Figure 7D). We then drove expression of DNK_v_4 only in motoneurons, using the *c164-Gal4* transgene. *c164-Gal4:UAS-DNK_v_4* flies displayed similarly low climbing scores compared with background controls (Figure 7D), suggesting that locomotion defects are due primarily to loss of K_v_4 function in motoneuron output. Our studies suggest that the adaptation and loss of reliable firing in DNK_v_4 motoneurons results in defective larval and adult locomotion.

Finally, we examined adult grooming to see if loss of K_v_4 function also affects other, non-locomotion, rhythmic behaviors. In these assays, flies were covered with a yellow dust, which triggered an immediate grooming behavior. After increasing times allowed for grooming, the fraction of “clean” flies were counted. Time-courses for grooming were performed for wild-type, the *UAS-DNK_v_4* background stock, and *elav-Gal4:UAS-DNK_v_4*. Similar to a previous study [37], nearly all wild-type flies were clean after two hours. In contrast, *elav-Gal4:UAS-DNK_v_4* flies only reached a 38% clean population in this time (Figure 7E). *elav-Gal4:UAS-DNK_v_4* flies, however, did groom, and for periods of time similar to wild-type, suggesting that their grooming behavior was defective. All of these behavioral studies suggest that loss of K_v_4 function, which is required for proper repetitive firing, results in defects in rhythmic behaviors known to depend on repetitive firing.

## Discussion

In this study, we eliminate function of Shal/K_v_4, and the entire somato-dendritic I_A_ current in *Drosophila* neurons, and perform a detailed characterization of the effects on neuronal firing. In single APs, we found that Shal/K_v_4 channels contribute to the repolarization, and afterhyperpoloarization, of the membrane. Similar findings were found for pyramidal neurons of CA1 [38] and the primary visual cortex [39] expressing a similar dominant-negative K_v_4.2 subunit. Most interesting, however, were the defects of DNK_v_4 neurons seen during prolonged depolarizations. DNK_v_4 neurons displayed a dramatically shortened delay to the first action potential (AP), a lower threshold for inducing repetitive firing, a higher initial firing frequency, and then a progressive decrement in AP amplitude to an adapted state. A role for Shal/K_v_4 currents regulating the latency to the first AP was also suggested in granule cells from the cerebellum [40] and in *Drosophila* larval motoneurons [27], [41]. Lowered threshold for repetitive firing, and an increase in firing frequency were seen in some mammalian neurons as well [38], [39].

### Absence of Cell Intrinsic Homeostasis in Cultured Embryonic Neurons

Studying the function of the somatic I_A_ current has been complicated in previous studies because multiple genes often underlie I_A_, there is a paucity of specific inhibitors available, and many systems exhibit electrical remodeling that occurs in response to genetic changes. For example, in the lobster stomatogastric ganglion, over-expression of *K_v_4*, regardless of whether the encoded channel is functional or not, leads to up-regulation of an I_h_ current [42]. Other studies using a targeted deletion of *K_v_4.2* (*K_v_4.2^−/−^*), have confirmed an important contributory role of K_v_4.2 to I_A_, but have also shown an incomplete loss of I_A_ [7]–[9]. Norris and Nerbonne (2010) have demonstrated that genetic deletion of K^+^ channel genes in *K_v_4.2^−/−^*, *K_v_4.3^−/−^*, and/or *K_v_1.4^−/−^* knock-out mice results in characteristic electrical remodeling of other K^+^ currents. In *Drosophila*, RNAi and pharmacological approaches to eliminate Shal/K_v_4 currents in identified neurons were also only partially successful at eliminating I_A_ [3], [4], [27], [41].

In this study, we eliminate Shal/K_v_4 function in *Drosophila* neurons by transgenic expression of a dominant-negative DNK_v_4 subunit. In cultured embryonic neurons, we observed the selective, and complete, loss of the somatic I_A_, with no change in the delayed rectifier K^+^ current, I_K_, encoded by *Shab/K_v_2* and *Shaw/K_v_3* [25], [28]. We also found no changes in I_Na_ or I_h_. These results are significant for two reasons. First, the selective effect of DNK_v_4 subunit expression on the Shal/K_v_4 current, and not on the currents encoded by Shab/K_v_2 and Shaw/K_v_3, strongly supports the long-standing hypothesis that K^+^ channel subunits will only multimerize within a subfamily, and not across subfamilies [11], [43]. Second, the lack of cell intrinsic electrical remodeling is unusual and has not been reported, to our knowledge, for any other cell type/preparation, and this has allowed us to identify effects on neuronal firing that are due exclusively to the loss of Shal/K_v_4 channels.

#### Why is there no cell intrinsic remodeling of K^+^ currents with the expression of DNK_v_4?

A recent report shows that a newly identified *Shal* mutant (*shal^495^*) exhibits an up-regulation of *Shaker* (*K_v_1*) RNA, and the presence of an A-type current recorded in cell bodies [23]. It is unclear why we did not observe similar electrical remodeling. One possibility is that cell intrinsic changes depend on alteration of the endogenous *Shal/K_v_4* gene, which occurs in the *shal^495^* mutant, but not in DNK_v_4 neurons. Another possibility is that cell intrinsic changes occur in DNK_v_4 neurons, but perhaps only in axons where we could not record, later in development, or only in subsets of neurons not recorded from in this study.

Cultured embryonic neurons are currently the only cells in *Drosophila* in which all of the K^+^ channels expressed have been genetically and electrophysiologically identified [25], [28], providing an excellent preparation for dissecting K^+^ channel function. In addition, embryonic neurons in primary cultures have been found to display structural and physiological properties similar to that expected in the nervous system, including presynaptic specializations, the expression of neurotransmitters, electrical activity, and synaptic plasticity [44]. Embryonic neurons in culture have also been used to study fast excitatory, and inhibitory, synaptic transmission, as well as plasticity in identified cells [45]–[48]. Our recordings from identified motoneurons in these cultures indeed displayed firing patterns very similar to those reported from the same larval motoneurons *in situ* [27], [41]. However, as with every cell culture system, extending conclusions to neurons, and behavior, *in vivo* is limited, and future studies will need to examine Shal/K_v_4 channel function in neurons *in vivo*, and preferably, from the same cells that drive the behavior being tested.

### Implications of Shal/K_v_4 Channel Regulation

One of the most interesting features of Shal/K_v_4 currents are their variable inactivation rates, as shown to span three orders of magnitude in *Drosophila* neurons [25], the regulation of these biophysical properties [31], [49], and their more recently reported activity-dependent local trafficking [50]. In this study, we examine whether these variable, regulated, properties of Shal/K_v_4 currents are likely to dictate the firing properties determined to be due to Shal/K_v_4 currents. Combined voltage- and current-clamp analysis of over 30 individual neurons from a wild-type population showed a direct correlation between these Shal/K_v_4 current properties and delay to the first AP and firing frequency; that is, greater Shal/K_v_4 current amplitudes, or slower inactivation rates, resulted in a longer delay to AP firing and a decreased firing frequency. Thus, regulation of Shal/K_v_4 channel properties by other proteins, or Shal/K_v_4 channel number by activity-dependent trafficking, is likely to translate into changes in neuronal firing output.

### How do Shal/K_v_4 Channels Maintain Excitability During Repetitive Firing?

Overall, the loss of K_v_4 function resulted in increased excitability of cells. With larger current injections, DNK_v_4 neurons fired quickly, with larger first APs and increased (initial) firing frequency, compared to wild-type. However, especially with increased current stimulation, DNK_v_4 neurons quickly adapted to a non-firing state, while wild-type neurons continued to fire throughout the stimulus. A very similar effect was seen in pyramidal neurons from the rat neonatal visual cortex expressing a similar dominant-negative K_v_4.2 subunit [39]. Although this appears to be a loss of excitability, the rapid accommodation is likely due to an “over-excited” state. One possibility is that the abnormally depolarized membrane prevents voltage-gated Na^+^ channels from fully recovering from inactivation. A similar model has been proposed for the I_A_ current encoded by K_v_3.4 in maintaining firing patterns in the lamprey spinal cord neurons which drive locomotion [51]. To test this possibility, we introduced a “pause” in the middle of the prolonged stimulus to DNK_v_4 neurons that allowed the membrane to repolarize, mimicking the probable role of Shal/K_v_4 channels. DNK_v_4 neurons did indeed regain the ability to fire. While an over-excited state could lead to a run-down of ionic gradients, or energy supply, these conditions would not be rescued by a “pause” on the order of milliseconds. Another possibility was that the properties governing recovery of Na^+^ channels from inactivation could have been altered. Careful analysis of the recovery of Na^+^ currents from inactivation in DNK_v_4 neurons, however, revealed no remodeling of these properties. We suggest a key role for K_v_4 channels in repolarization of the membrane for Na^+^ channel recovery, and reliable repetitive firing.

### Role of Shal/K_v_4 Channels in Rhythmic Behavior

Reliable repetitive firing is likely to be extremely important in rhythmic behaviors, like crawling, walking, and grooming. These behaviors depend on three types of neurons: central pattern generators (CPGs), motoneurons, and sensory neurons. When we expressed DNK_v_4 throughout the entire nervous system, we found that indeed animals displayed locomotion and grooming defects compared to wild-type, indicating the significance of Shal/K_v_4 function in repetitive firing. Prolonged stimuli used in this study evoked bursts of firing similar to motoneuron activity seen during locomotion. To test whether the primary role of Shal/K_v_4 channels for locomotion is in motoneurons, we first recorded from identified wild-type and DNK_v_4 motoneurons, then knocked-out Shal/K_v_4 function specifically in motoneurons in the whole animal. The loss of K_v_4 function in motoneurons impaired excitability during repetitive firing similar to other neurons, suggesting that signaling to muscles was likely to be defective. Additionally, in repeated, behaviorally relevant pulses of depolarization, identified motoneurons indeed more easily adapted during these bursts of AP firing. Interestingly, loss of excitability in motoneurons was not as severe as compared to other neurons. Because the importance of reliable repetitive firing in motoneurons, it is possible that they have additional mechanisms to safeguard firing properties.

When Shal/K_v_4 channel function was knocked-out specifically in motoneurons in the whole animal, larvae and adults displayed obvious locomotion defects compared to wild-type controls. Indeed, larval crawling speeds and adult climbing were impaired as severely as when DNK_v_4 was expressed throughout the nervous system. These results suggest that Shal/K_v_4 channel regulation of repetitive firing in motoneurons is required for normal rhythmic behavior. Since flight requires sustained high frequency firing, it is likely that defects in flight may even be more dramatic. Shal/K_v_4 channels, however, may also be important for signaling in other upstream cells in the locomotion circuitry, such as in CPG neurons. Since the CPG neurons have not been identified though, future studies will need to investigate this possibility. Recently, the importance of PNS feedback from identified multi-dendritic (md) sensory neurons has been shown to play a critical role in larval locomotion [52]–[54]. It is thought that sensory input detected by dendrites of md neurons in the PNS is communicated to CPG neurons in the CNS that then drive motoneurons and ultimately, motor output. When sensory input from md neurons was conditionally silenced, firing patterns from motoneurons [53] and peristaltic waves in larvae were altered [54]. Indeed, when we expressed DNK_v_4 only in these md neurons, using the *109(80)-Gal4* driver, larval locomotion speeds were also significantly slowed compared to background controls. It is therefore possible that Shal/K_v_4 channels also function in the sensory feedback part of the locomotor circuitry, not likely as a sensor, but as an integrator of signals in dendrites.

Thus, in addition to a role in the firing output of cells, Shal/K_v_4 channels, which are localized exclusively to somato-dendritic compartments of cells [23], [24], may function in the dendrites of motoneurons and sensory neurons, modulating the integration of post-synaptic potentials. Recent work has indeed highlighted the role of mammalian K_v_4 channels in dendritic excitability [7], [38], [50], [55]–[59]. Since Shal/K_v_4 channels can exhibit variable properties from cell to cell, Shal/K_v_4 channels may play different roles among different motoneurons. Indeed, differences in the intrinsic electrical properties of larval motoneurons with different size synaptic terminals have also begun to be explored [27]. Future studies will need to integrate the biophysical regulation, subcellular localization, and activity-dependent local trafficking of Shal/K_v_4 channels to gain a full understanding of Shal/K_v_4 channel function in locomotion and other rhythmic behaviors.

## Materials and Methods

### Fly Stocks and Transgenic Lines Generated

Canton-S and *w^1118^ Drosophila* strains were used as wild-type in this study. The *elav-Gal4* line was obtained from the Bloomington *Drosophila* Stock Center (Indiana University, Bloomington, IL), the *c164-Gal4* line [60] was obtained from Dr. Vivian Budnik, the *RRa-Gal4, UAS-mCD8-GFP* line [32], [33] was generated and obtained from Dr. Miki Fujioka, and the 109(80)-Gal4 line [61] was obtained from Dr. Yuh Nung Jan. For transgenic *UAS-DNK_v_4* lines, the *Shal2* cDNA was modified to contain an HA tag (YPYDVPDYA) fused to the N-terminus, and encode a phenylalanine at amino acid 362, instead of the conserved tryptophan residue. This modified *Shal2* was subcloned into the *pUAST* transformation vector; generation of this construct was performed by GenScript, Inc. (Piscataway, NJ). Microinjection into embryos, and generation of transgenic lines were performed by standard procedures.

### Embryonic Cell Cultures

Embryos aged 5–6 hrs (25°C, stage 9–10) were dissociated into culture media, as previously described [25], [28], [31]. Immunostaining of cultures was performed as previously described [31]. Anti-GFP (Torrey Pines Biolabs, East Orange, NJ) and anti-HA (Covance Research Products, Emeryville, CA) was used at 1∶1000 and 1∶100, respectively; FITC and rhodamine conjugated secondary antibodies (Jackson ImmunoResearch Laboratories, West Grove, PA) were used at 1∶500.

### Electrophysiology

All whole-cell recording was done in the perforated patch configuration, using 400 ug/mL Amphotericin-B (Sigma-Aldrich) in the patch pipette, as described previously [31]. Cell cultures were 3 to 4 days old. For recording K^+^ currents, we used a K-internal solution of KCl (in mM), 140; MgCl_2_, 2; EGTA, 11; HEPEs, 10; pH 7.2, and a Choline-external solution of Choline-Cl (in mM), 140; KCl, 2; MgCl_2_, 6; HEPEs, 5, pH 7.2. For recording only Na^+^ currents, we used a Cs-internal solution of CsCl (in mM), 140; CaCl_2_, 0.1; MgCl_2_, 2; EGTA, 1.1; HEPEs, 10, pH 7.2 with NaOH, and external solution of NaCl (in mM), 140; CsCl, 2; MgCl_2_, 6; HEPEs, 5, pH 7.2. For current-clamp recordings, we used an internal solution of K-Gluconate (in mM), 120; KCl, 20; HEPEs, 10; EGTA, 1.1; MgCl_2_, 2; CaCl_2_, 0.1, pH 7.2, and external solution of NaCl (in mM), 140; KCl, 2; MaCl_2_, 6; HEPEs, 5, pH 7.2. Electrode resistances for all voltage and current-clamp experiments were 3–8 MΩ. Gigaohm seals were obtained for whole-cell recording. Cell capacitances recorded from wild-type and DNK_v_4 neurons were also similar (2.3±0.2 pF and 2.5±0.3 pF for wild-type (N = 15) and DNK_v_4 (N = 15), respectively). Data was recorded using an Axopatch200B amplifier (Molecular Devices Corp.). Recordings were digitized at 5 kHz and filtered at 2 kHz, using a lowpass Bessel filter. Averaged data presented as mean ± SEM; *p<0.05, **p< 0.01, *** p<0.001 (student's t test).

### Behavioral Assays

For larval crawling assays, age-synchronized embryos were collected from flies for 24 hours, then incubated at 25°C for 4 additional days. Third instar larvae of similar size were briefly rinsed in dH_2_O, then individually placed in the center of 90 mm 1% agarose plates set over a 0.5×0.5 cm square grid. The number of squares crossed, in a 5 minute period, were recorded for each larva.

For adult locomotion, bottles of flies were cleared and newly-eclosed flies were collected over 24 hours and aged for an additional 48 hours at 25°C. In each test, modeled after previous studies [35], [36], 35 males in a 12.4 cm tall tube were allowed to climb upwards for 30 seconds into a second tube inverted on top of the first. The flies that successfully climbed into the second tube were given 30 seconds to climb from the bottom of the tube into a third tube. This process was continued through ten successive tubes.

For grooming assays, flies were collected and aged similar to adult locomotion. Groups of 20–30 flies were “dusted” with Reactive yellow 86 dust (Organic Dyestuffs Corp., East Providence, RI) for 10 seconds, then moved to a clean vial. Dust was baked at 50°C overnight, sifted through chessecloth mesh, and 30 mg was transferred to a vial for each assay. Assay was modified from a previous study [37].

All averaged data presented as mean ± SEM; *p<0.05, **p<0.01 (student's t test).

## Supporting Information

Figure S1 **No Change in the Steady-State Inactivation Properties of I_Na_ in DNK_v_4 Neurons.** *A*, Representative voltage-clamp recordings from wild-type (wt) and DNK_v_4 (DN) neurons in response to a test voltage-jump to −10 mV, following a 500 ms pre-pulse voltage of −100 to −20 mV, in 5 mV increments. *B*, Steady-state inactivation plot of normalized peak Na^+^ current versus pre-pulse potential; shown are the averaged points of 13 wt and 5 DN neurons. For each cell, points were fit with a single Boltzmann equation: I/I_max_ =  [1+ exp(V-V_1/2_)/k]^−1^. The average pre-pulse potential at which half the channels are inactivated (V_1/2_) was not significantly different between wild-type (−48.3±5.1 mV) and DN (−55.6±3.1 mV); k values were also not significantly different (6.1±0.65 for wt, 6.2±0.64 for DN).
(TIF)

Figure S2 **Pre-pulse Current Injection Regulates the Latency to AP Firing.** *A*, Representative wild-type (wt) and DNK_v_4 (DN) voltage responses to a 500 ms “test-injection” of 100 pA, following a 500 ms “pre-injection” of −30, 0, or +30 pA. Pre-injection of −30 pA resulted in the greatest delay to AP firing during the test-injection. In wt, increasing the pre-injection current to 0 and +30 pA, resulted in a shorter and shorter delay to firing; the +30 pA pre-injection resulted in a latency similar to DN neurons. *B*, We also varied the duration of the −30 pA and +30 pA pre-injections from 100 ms to 1 s. Average latency times are plotted for each condition (N = 7 for wt, N = 9 for DN). Note that the latency to AP firing correlated with conditions that gave a longer hyperpolarizing membrane potential; that is, with longer duration hyperpolarization, or shorter duration depolarization, the delay to AP firing was prolonged. These experiments were also performed with DN neurons, which showed very little difference in the delay to AP firing with different pre-injections, confirming that in wild-type, the dependence of this delay on membrane voltage is likely to act nearly entirely on K_v_4 channels.
(TIF)
