# Supplementary figures and images for: Shal/Kv4 Channels Are Required for Maintaining Excitability during Repetitive Firing and Normal Locomotion in Drosophila
Source: PLoS One. 2011 Jan 17;6(1):e16043. doi: 10.1371/journal.pone.0016043 (PMC3022017; doi:10.1371/journal.pone.0016043)

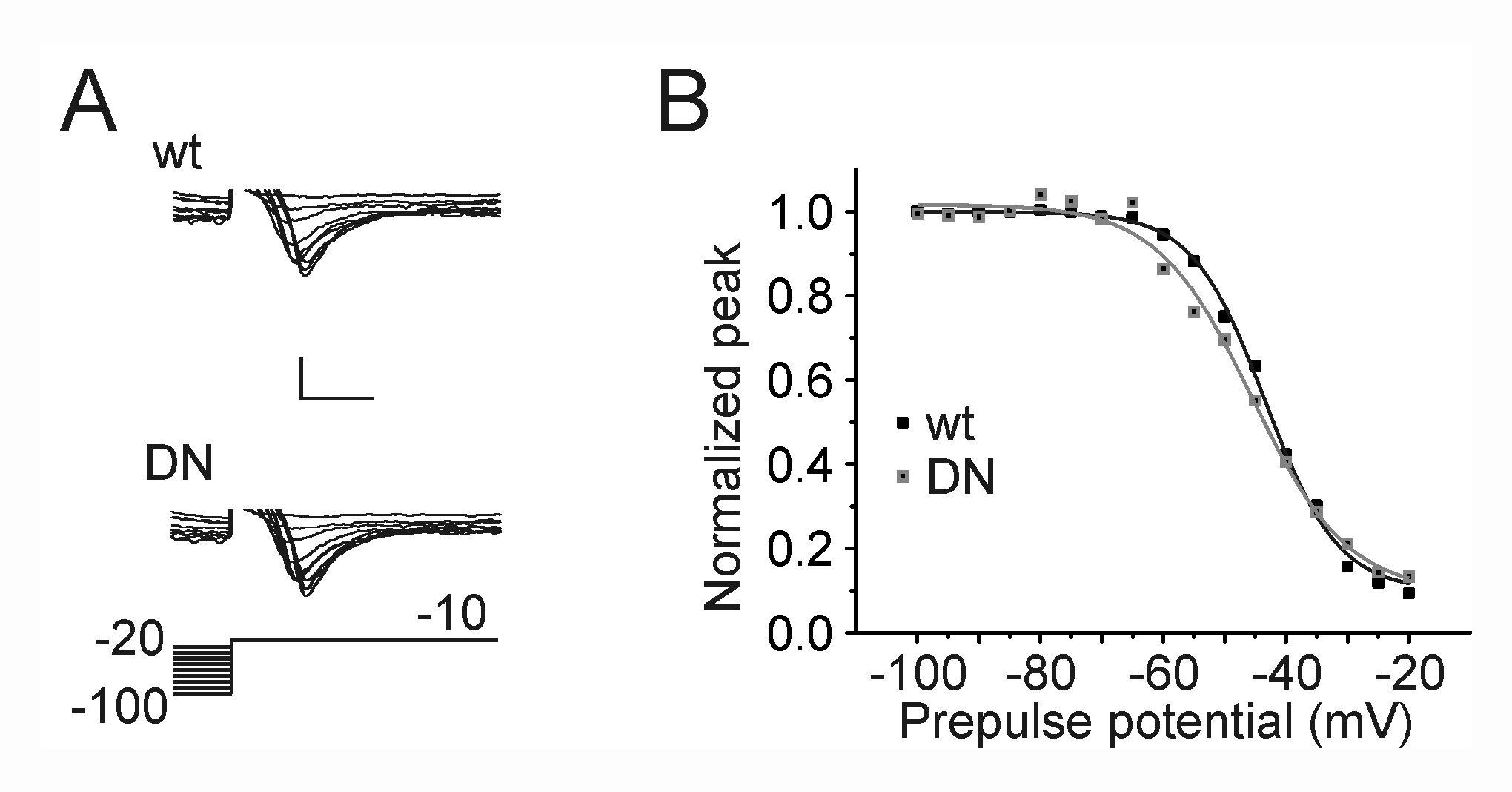

Supplement: Figure S1 — No Change in the Steady-State Inactivation Properties of INa in DNKv4 Neurons. A, Representative voltage-clamp recordings from wild-type (wt) and DNKv4 (DN) neurons in response to a test voltage-jump to −10 mV, following a 500 ms pre-pulse voltage of −100 to −20 mV, in 5 mV increments. B, Steady-state inactivation plot of normalized peak Na+ current versus pre-pulse potential; shown are the averaged points of 13 wt and 5 DN neurons. For each cell, points were fit with a single Boltzmann equation: I/Imax = [1+ exp(V-V1/2)/k]−1. The average pre-pulse potential at which half the channels are inactivated (V1/2) was not significantly different between wild-type (−48.3±5.1 mV) and DN (−55.6±3.1 mV); k values were also not significantly different (6.1±0.65 for wt, 6.2±0.64 for DN). (TIF) [file pone.0016043.s001.tif]

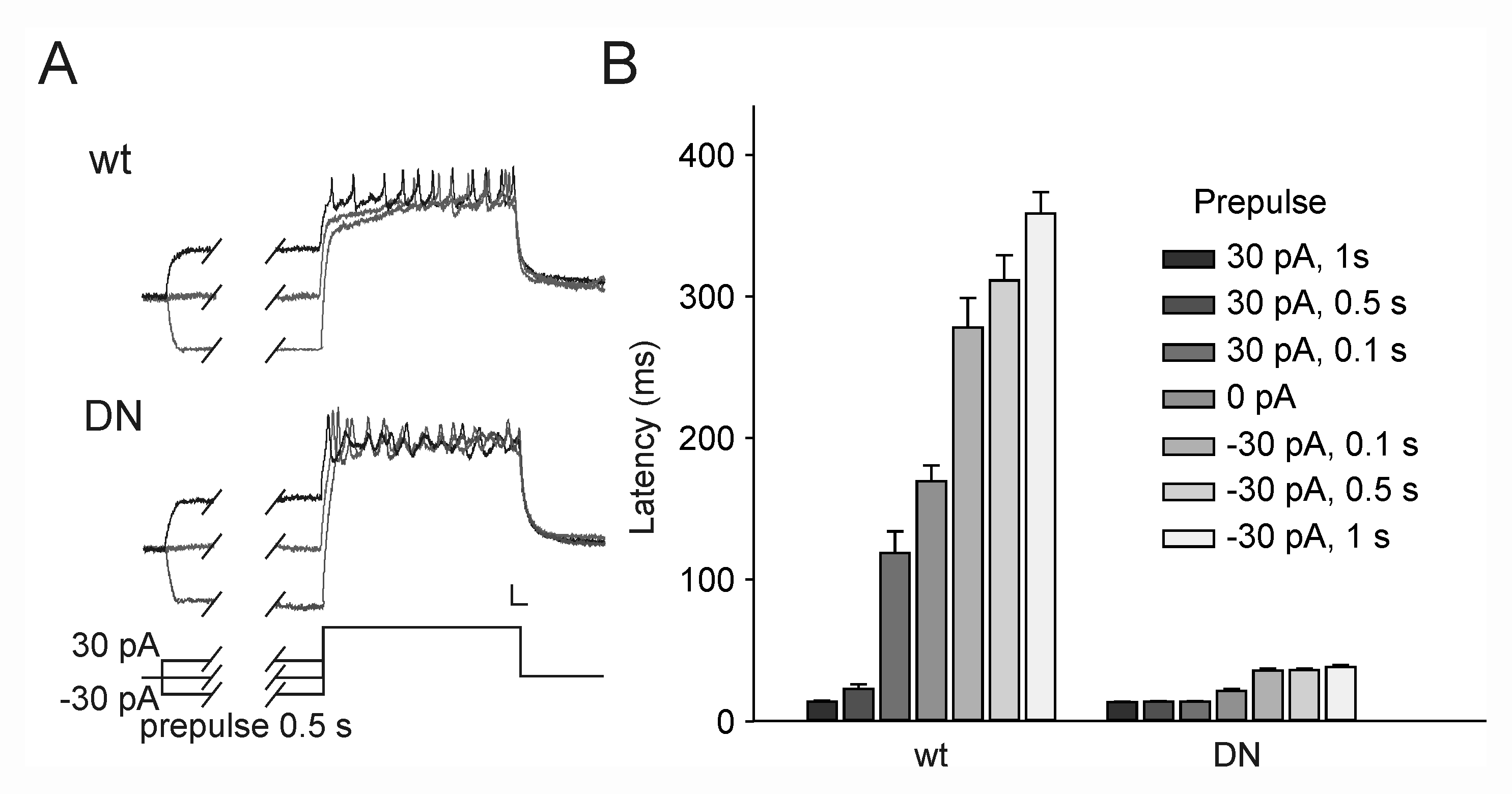

Supplement: Figure S2 — Pre-pulse Current Injection Regulates the Latency to AP Firing. A, Representative wild-type (wt) and DNKv4 (DN) voltage responses to a 500 ms “test-injection” of 100 pA, following a 500 ms “pre-injection” of −30, 0, or +30 pA. Pre-injection of −30 pA resulted in the greatest delay to AP firing during the test-injection. In wt, increasing the pre-injection current to 0 and +30 pA, resulted in a shorter and shorter delay to firing; the +30 pA pre-injection resulted in a latency similar to DN neurons. B, We also varied the duration of the −30 pA and +30 pA pre-injections from 100 ms to 1 s. Average latency times are plotted for each condition (N = 7 for wt, N = 9 for DN). Note that the latency to AP firing correlated with conditions that gave a longer hyperpolarizing membrane potential; that is, with longer duration hyperpolarization, or shorter duration depolarization, the delay to AP firing was prolonged. These experiments were also performed with DN neurons, which showed very little difference in the delay to AP firing with different pre-injections, confirming that in wild-type, the dependence of this delay on membrane voltage is likely to act nearly entirely on Kv4 channels. (TIF) [file pone.0016043.s002.tif]
